# Functional relevance of the extrastriate body area for visual and haptic object recognition: a preregistered fMRI-guided TMS study

**DOI:** 10.1093/texcom/tgad005

**Published:** 2023-04-13

**Authors:** Hicret Atilgan, J X Janice Koi, Ern Wong, Ilkka Laakso, Noora Matilainen, Achille Pasqualotto, Satoshi Tanaka, S H Annabel Chen, Ryo Kitada

**Affiliations:** Psychology, School of Social Sciences, Nanyang Technological University, 48 Nanyang Avenue, Singapore 639818, Singapore; Psychology, School of Social Sciences, Nanyang Technological University, 48 Nanyang Avenue, Singapore 639818, Singapore; IMT School for Advanced Studies Lucca, Piazza S. Francesco, 19, 55100 Lucca LU, Italy; Department of Electrical Engineering and Automation, Aalto University, Otakaari 3, 02150 Espoo, Finland; Department of Electrical Engineering and Automation, Aalto University, Otakaari 3, 02150 Espoo, Finland; Faculty of Human Sciences, University of Tsukuba, 1-1-1 Tennodai, Tsukuba, Ibaraki 305-8577, Japan; Department of Psychology, Hamamatsu University School of Medicine, 1-20-1 Handayama, Higashi Ward, Hamamatsu, Shizuoka 431-3192, Japan; Psychology, School of Social Sciences, Nanyang Technological University, 48 Nanyang Avenue, Singapore 639818, Singapore; Centre for Research and Development in Learning, Nanyang Technological University, 61 Nanyang Drive, Singapore 637335, Singapore; Lee Kong Chian School of Medicine (LKCMedicine), Nanyang Technological University, 11 Mandalay Road, Singapore 308232, Singapore; Psychology, School of Social Sciences, Nanyang Technological University, 48 Nanyang Avenue, Singapore 639818, Singapore; Centre for Research and Development in Learning, Nanyang Technological University, 61 Nanyang Drive, Singapore 637335, Singapore; Graduate School of Intercultural Studies, Kobe University, 1-2-1 Tsurukabuto, Nada Ward, Kobe, Hyogo 657-0013, Japan; Lee Kong Chian School of Medicine (LKCMedicine), Nanyang Technological University, 11 Mandalay Road, Singapore 308232, Singapore

**Keywords:** body, EBA, haptics, TMS, tool

## Abstract

The extrastriate body area (EBA) is a region in the lateral occipito-temporal cortex (LOTC), which is sensitive to perceived body parts. Neuroimaging studies suggested that EBA is related to body and tool processing, regardless of the sensory modalities. However, how essential this region is for visual tool processing and nonvisual object processing remains a matter of controversy. In this preregistered fMRI-guided repetitive transcranial magnetic stimulation (rTMS) study, we examined the causal involvement of EBA in multisensory body and tool recognition. Participants used either vision or haptics to identify 3 object categories: hands, teapots (tools), and cars (control objects). Continuous theta-burst stimulation (cTBS) was applied over left EBA, right EBA, or vertex (control site). Performance for visually perceived hands and teapots (relative to cars) was more strongly disrupted by cTBS over left EBA than over the vertex, whereas no such object-specific effect was observed in haptics. The simulation of the induced electric fields confirmed that the cTBS affected regions including EBA. These results indicate that the LOTC is functionally relevant for visual hand and tool processing, whereas the rTMS over EBA may differently affect object recognition between the 2 sensory modalities.

## Introduction

Studies on the mechanism underlying sensory processing have generally agreed that visual input is processed in a parallel-distributed manner (Zeki 1998) and then integrated to represent objects in the brain. In contrast, it is not fully understood whether a similar framework can be applied to nonvisual sensory modalities such as touch (Kitada 2016; Sathian 2016). In the present study, we focused our investigation on the involvement of the lateral occipito-temporal cortex (LOTC) in haptic and visual object recognition.

Previous studies have identified the functionally specialized regions for visual object recognition in LOTC. For instance, the extrastriate body area (EBA) is more sensitive to visually perceived human body parts than other categories of objects (Downing et al. 2001; Taylor et al. 2007; Chan et al. 2010). EBA is closely located to and partially overlapped with other functionally specialized regions, such as the motion-sensitive region (hMT+; Weiner and Grill-Spector 2011; Ferri et al. 2013), object-sensitive region (LO; Grill-Spector et al. 2001), and a region that is more sensitive to tools (e.g. scissors, abacus, and combs) than other object categories (Bracci et al. 2010, 2012; Bracci and Peelen 2013; Peelen et al. 2013). A more recent study demonstrated that activation patterns in LOTC representing the hand prostheses (tools) and actual hands differed more distinctly in prosthesis users than in control participants, suggesting an interaction of multiple specialized regions within LOTC (Maimon-Mor and Makin 2020).

Another line of studies has shown that LOTC is sensitive to signals of nonvisual sensory modalities (Amedi et al. 2001, 2002; James et al. 2002; Astafiev et al. 2004; Ricciardi et al. 2007, 2009; Stilla and Sathian 2008; Peelen et al. 2013; Striem-Amit and Amedi 2014). More importantly, functionally specialized regions in LOTC show similar activity preferences in both sighted and congenitally blind individuals (for review, see Ricciardi and Pietrini 2011). For instance, functional MRI studies have shown that EBA is more sensitive to haptically perceived body parts than other objects (Kitada et al. 2009, 2014; Costantini et al. 2011). Several studies also observed such body-sensitive activity in early blind individuals (Kitada et al. 2014; Striem-Amit and Amedi 2014), suggesting that EBA is involved in the supramodal representation of human bodies. Thus, LOTC may contain critical nodes of the networks for modality-independent object representation.

One of the limitations of these neuroimaging studies is that they can provide correlational inferences but not necessarily causal inferences between task and neural activity. Alternative approaches such as brain stimulation can complement the above-mentioned neuroimaging findings. For example, previous studies have shown that application of repetitive transcranial magnetic stimulation (rTMS) to EBA impaired visual identification of body parts but not objects in other categories (Urgesi et al. 2004; Pitcher et al. 2009). Moreover, rTMS to LO increased the reaction time for the visual discrimination of object forms but not object orientations (Chouinard et al. 2017). These findings confirm the functional relevance of EBA and LO for visual object processing.

However, there are 2 issues that remain unclear. First, though regions in and around EBA are sensitive to tools (Bracci et al. 2010, 2012; Peelen et al. 2013), this region’s functional relevance in tool representation is unclear. A previous study demonstrated that transcranial magnetic stimulation (TMS) over left LOTC delayed the response time (RT) for judgment of actions associated with tools relative to the RT for judgment of the tool locations (Perini et al. 2014). However, it is still unknown if this region is functionally more relevant for the recognition of tools than other categories of objects. Second and more importantly, the extent to which LOTC is critical for nonvisual object processing is still under debate. Some studies have demonstrated that rTMS over hMT+ impaired task performance of tactile motion judgments (Ricciardi et al. 2011; Basso et al. 2012; Amemiya et al. 2017) and that rTMS over LOTC affected visual and tactile illusions (Mancini et al. 2011). In contrast, rTMS over LO caused no behavioral impairment, although a transient change in LO activity was observed in fMRI (Kassuba et al. 2014). Snow et al. (2015) demonstrated that a patient with lesions in the bilateral occipito-temporal cortex has a deficit in visual, but not haptic, object recognition (Snow et al. 2015). To our best knowledge, the functional relevance of EBA in nonvisual body processing has not been investigated.

In the present study, we conducted a preregistered fMRI-guided rTMS experiment to examine whether EBA is functionally relevant for hand and tool recognition regardless of the sensory modalities. We adopted an offline rTMS paradigm in which continuous theta-burst stimulation (cTBS, Huang et al. 2005) was applied to either left EBA, right EBA, or vertex in separate sessions. Before and after cTBS, the participants used either vision or touch to identify 3 categories of objects: hands, teapots (tools), and model cars (control objects). Because cTBS can cause an inhibitory effect in the target region (Huang et al. 2005; Rossi et al. 2009), we predicted that rTMS over the unilateral EBA would decrease performance for the identification of hands and teapots as compared with cars, regardless of the sensory modality.

## Materials and methods

### Participants

We recruited 33 right-handed participants for this experiment, taking possible dropouts into account. Seven participants withdrew from the study because of incidental findings, failure in EBA localization, rTMS-associated discomfort, or schedule conflicts. The remaining 26 healthy sighted volunteers (17 male; age range: 21–38 years; mean ± SD age = 26 ± 3.9 years) completed the experiment. All participants provided written informed consent prior to the experiment. All methods were carried out in accordance with the approved safety guidelines (Rossi et al. 2009) and the Declaration of Helsinki. Participants’ handedness was confirmed by the Fazio Handedness Inventory (Fazio et al. 2013), a revised questionnaire of the Edinburgh Inventory (Oldfield 1971). The study protocol was reviewed and approved by the Institutional Review Board at Nanyang Technological University, Singapore (NTU IRB-2018-07-017). All of the participants had normal or corrected-to-normal vision, and none were on any medication or had a history of neurological disorder or psychiatric illness, drug or alcohol abuse. All participants were paid for their time.

We estimated the sample size with G*Power 3 (Faul et al. 2007) based on the effect size from previous studies using rTMS (*d* = 0.75 ± 0.63, mean ± SD) and our pilot experiment with 6 participants who were not recruited for the main experiment (*d* = 0.66). The minimum number of participants (21) was preregistered at the Open Science Framework (https://osf.io/xudj6). We recruited more than 21 participants to further minimize type II error. The decision to stop the recruitment of the participants was independent of the collected data, because the analysis was not initiated until all data had been gathered.

The participants went through 5 sessions. Each participant participated in an fMRI session to localize EBA in the first session; their resting motor threshold was measured and behavioral training was conducted in the second session; and finally, the participants completed the 3 rTMS sessions.

### fMRI localization of target regions

#### Scanning parameters

A 3 Tesla whole-body MRI scanner (Siemens Magnetom PRISMA) equipped with a 12-channel head coil at the Cognitive Neuroimaging Centre located at NTU, Singapore, was used to acquire T2*-weighted functional images, as well as T1- and T2-weighted high-resolution anatomical images for each participant. A multiband echo-planar imaging sequence was used for T2*-weighted functional image acquisition with the following parameters: TR = 1,000 ms, echo time (TE) = 38 ms, flip angle = 55°, 330 slices with 3 mm slice thickness, 39 axial slices, field-of-view = 220 × 220 mm^2^, in-plane resolution = 2.97 × 2.97 mm^2^, and MB factor = 3. T1 anatomical images were acquired using a T1-weighted 3D magnetization prepared rapid acquisition gradient echo sequence with the following parameters: voxel size 1 × 1 × 1 mm^3^, TR = 2,250 ms, TE = 2.07 ms, and flip angle = 9°. T2 anatomical images were acquired using a T2-weighted echo-planar fast spin echo sequence with the following parameters: voxel size 1 × 1 × 1 mm^3^, TR 3,200 ms, TE = 410 ms, and flip angle = 120°. T1- and T2-weighted images were used for localization of EBA and computer simulation of electric fields (EFs) induced by rTMS.

#### fMRI task design

We adopted a standard task design to localize EBA (Downing et al. 2001; Kitada et al. 2014; Okamoto et al. 2014). The visual stimuli were monochromatic images of body parts, teapots, cars, and textures (Fig. 2a). The mean luminance and size of images were matched between object categories with Adobe Photoshop software (Adobe, San Jose, CA, USA). The experiment consisted of 3 functional runs, with each run having 21 blocks and each block lasting 15 s. A 15-s fixation-only baseline condition was added before the first baseline block (15 s × 21 blocks + 15 s baseline = 330 s, 330 volumes in total). The 1st, 6th, 11th, 16th, and 21st blocks were fixation-only baseline conditions. Each block other than the baseline-condition blocks was made of 20 images of the same category, with each image lasting 300 ms and the interstimulus-intervals (ISIs) being 450 ms. Participants were instructed to fixate on the white cross and respond to the red cross, which appeared twice in each block, by pressing the button with their right thumb. The block for each category was repeated 4 times and counterbalanced across runs and participants. The Presentation software (Neurobehavioral Systems, Berkeley, CA, USA) was used to present the stimuli and collect responses.

#### Image preprocessing and analysis

fMRI data were analyzed with SPM12 (Welcome Trust Centre for Neuroimaging, London, UK; RRID: SCR_007037), implemented in MATLAB R2018a (MathWorks, Natick, MA, USA). For each participant, the first 15 functional images were discarded to allow the MR signal to reach a state of equilibrium. To correct for head motion, the remaining volumes (315 volumes per run) from each run were realigned to the first image and again realigned to the mean image after the first realignment. The T1-weighted anatomical image was co-registered to the mean of all realigned images. Each co-registered T1-weighted anatomical image was normalized to the Montreal Neurological Institute (MNI) T1 image template (ICBM 152). The parameters from this normalization process were then applied to each functional image. Finally, the spatially normalized functional images were filtered using a Gaussian kernel of 4-mm full-width at half-maximum in the *x*-, *y*-, and *z*-axes.

We used a general linear model for the statistical analyses. Blood-oxygen-level-dependent signal was modeled with boxcar functions that were convolved with the canonical hemodynamic response function. A design matrix consisted of 3 functional runs of each participant with 4 task-related regressors (body parts, cars, teapots, and textures) and 6 nuisance motion parameters. Motion parameters (3 displacements and 3 rotations) were obtained from the rigid-body realignment. The time series for each participant was high-pass-filtered at 1/128 Hz. Temporal autocorrelations were modeled and estimated from the pooled active voxels by the FAST model and were used to whiten the data (Corbin et al. 2018; Olszowy et al. 2019). We used linear contrasts to localize EBA, tool-sensitive region, and LO. The resulting set of voxel values for each contrast constituted the SPM{t}.

Individual localization of TMS targets was conducted in MNI space to ensure that the targets were sufficiently close to previously reported regions (Kitada et al. 2014). More specifically, to define each participant’s EBA, we compared the category of bodies with the mean of other object categories (Peelen and Downing 2005a, 2005b). The threshold for the SPM{t} (i.e. height threshold) was set at *P* < 0.001 uncorrected for multiple comparisons. To define the target area within EBA in each participant, we used 2 additional criteria. First, we chose the peak coordinates that were in close proximity to the previously reported foci of EBA that respond to multisensory inputs (Kitada et al. 2014). Second, we chose the peak coordinates that were located close to the skull such that the EF induced by rTMS covered the target area.

As a control region, we used the vertex. This decision was made for 2 reasons. First, the vertex is often used as a control region in TMS studies (e.g. Jung et al. 2016) and is distant from EBA. Second, in our previous fMRI study, activity around the vertex was comparable between perceived hands and other objects (Kitada et al. 2014). The vertex was anatomically defined as the intersection between the central sulcus and interhemispheric fissure for each participant. Finally, we transformed the MNI coordinates for EBA and control regions back to each participant’s native brain space. These regions of interest were used as targets of rTMS.

We then depicted group-average activation in EBA and LO localizers and teapot-sensitive activation. We used the same contrast as above to depict EBA, whereas we localized LO for each participant by comparing objects (body parts, teapots, and cars) with textures and teapot-sensitive activation by comparing teapots with cars. We performed 1-sample *t*-tests on the contrast estimates that were obtained from the individual analyses.

After depicting regions for EBA, LO, and teapot-sensitive activation, we performed the conjunction analysis using an inclusive masking procedure (Rajaei et al. 2018; Kitada et al. 2021). This approach is similar to the standard conjunction analysis, as the whole brain was used as the search volume for the overlap of activation. In all fMRI analyses, the statistical threshold for the spatial extent test on the clusters was set at *P* < 0.05, family-wise error (FWE) corrected for multiple comparisons. The height (cluster-forming) threshold was set at *P* < 0.001 (uncorrected). This threshold is sufficiently high to use the random-field theory to control FWE rate (Flandin and Friston 2019). We used FreeSurfer (version 7.1.1) on Neurodesk (https://www.neurodesk.org/) for data presentation.

### TMS

We used the cTMS (Rogue Research, Montreal, Canada) with a figure-eight coil (70 mm diameter, Rogue Research) and the Brainsight TMS Navigation system (Rogue Research). We used one of the standard stimulation protocols of the cTMS, which generates a unidirectional biphasic pulse with a positive duration of 60 μs, a negative duration of 185 μs in dI/dt (A/us), and a ratio of the peak of the negative phase to the positive phase (m ratio) of 0.225. We employed an offline TMS paradigm.

#### The definition of TMS intensity and stimulation protocol

We defined the intensity of TMS for each participant based on the resting motor threshold. We anatomically defined the hand area of the left primary motor cortex (M1) and applied TMS over the region to record motor-evoked potentials (MEPs) from the right first dorsal interosseus through electrodes with the BrainSight EMG isolation unit. The coil was positioned over the corresponding motor area at an angle of 45 degrees, with the coil cable pointing in the anterior–posterior direction (i.e. posterior–anterior-induced current). Resting motor threshold was defined as the lowest TMS intensity at which over 5 out of 10 MEPs were over 50 μV amplitude (Rossini et al. 1994, 2015).

We used the standard cTBS protocol (Huang et al. 2005; Rossi et al. 2009), which consisted of 3 bursts of pulses given at 50 Hz, repeated every 200 ms with a frequency of 5 Hz (600 pulses in total for 40 s). The intensity was fixed at 70% of the resting motor threshold. Like previous studies on TMS over EBA, TMS was delivered with the coil cable pointing upwards and parallel to the midline (Pitcher et al. 2009). We stimulated the vertex with the coil cable pointing backward and parallel to the midline.

#### Experimental design

**Fig. 1:**
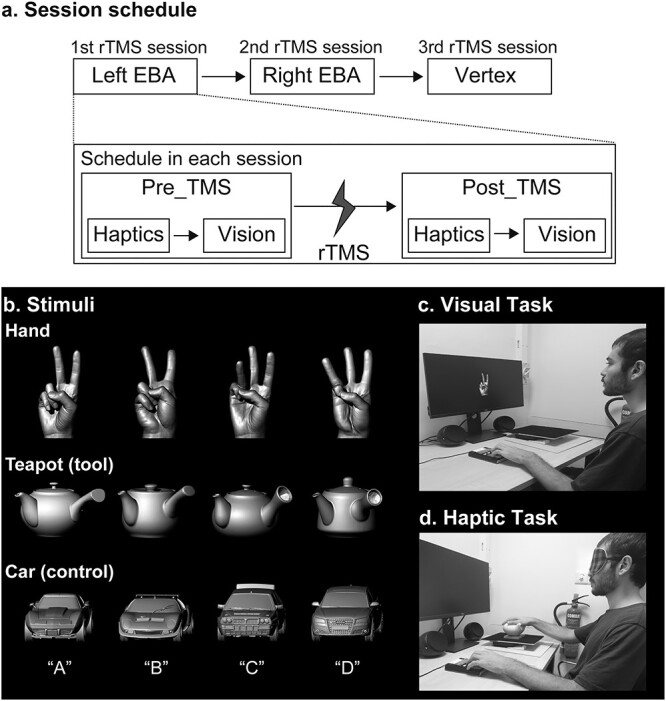
Session schedule and stimulus presentation a) session schedule. The participants went through 3 rTMS sessions, each targeting a specific site determined using fMRI localizer task. Within each session, the participants performed haptic and visual tasks separately. We designed to counterbalance the order of stimulation sites and sensory modalities across participants. Before these sessions, participants completed practice trials until achieving a minimum accuracy of 70% in each sensory modality. b) Stimuli. We used 3 categories of objects: hands, teapots (tool), and cars (control) as in the previous fMRI study (Kitada et al. 2014). One of the 4 letters (A–D) was assigned to each exemplar within each object category. Participants identified the exemplars by pressing one of the 4 buttons corresponding to the letters. The sizes of stimuli were changed for the purpose of the presentation. c) Visual task. Monochromatic images of the stimuli were shown on a monitor screen, and participants used their left hand to press the button. d) Haptic task. Blindfolded participants explored the stimuli with their right hand and gave a button response with the left hand.

The experiment had 4 within-subject factors: (i) target site (left EBA, right EBA, and vertex), (ii) object (hand, teapot, and model car), (iii) phase (pre_TMS and post_TMS), and (iv) sensory modality (vision and haptics). The participants went through 3 sessions on separate days, each of which involved only 1 target site (Fig. 1a). Sessions were separated within participants by at least 4 days [session interval, 10.86 ± 6.2 days (mean ± SD)] to control for carry-over effects because session intervals of <24 h have been shown to result in cumulative effects (Maeda et al. 2002; Bäumer et al. 2003).

We designed to counterbalance the order of the target regions and the order of the sensory modality across participants. The order of the sensory modality was held constant within participants; if the participant started vision first in the pre_TMS phase, they started vision first in the post_TMS phase. However, 14 started haptics first and 12 started vision first because of the dropouts of the participants. The average order of the target regions (1 for the first cTBS session; 3 for the last cTBS session) was roughly matched: 2.1 for left EBA, 1.9 for right EBA, and 2.0 for the vertex.

#### Stimuli/object categories

We used the same object categories as in our previous fMRI study (Kitada et al. 2014): hands, teapots, and model cars (Fig. 1b). Teapots were used as tools for the following 2 reasons. First, they are hand-sized and familiar objects. Second, teapot is similar to objects categorized as tools in previous studies: abacus, brush, key, button, pens, combs, and forks (Bracci and Peelen 2013; Peelen et al. 2013). We assumed that teapot is a part of everyday “act-with” objects that effectively activate LOTC (Bracci and Peelen 2013). Model cars were used as the control object. All objects were created with a 3D printer (Connex 500; Stratasys, MN, USA) after all objects and an actor’s hand shapes were scanned with a 3D digitizer (MH, Artec Group, Luxembourg for the hand stimuli; ATOS, GOM, Germany for the objects). Each category contained 4 exemplars that were named A–D (Fig. 1b). The exemplars of hands differed in (i) identity and (ii) actions (2 vs. 3) to match difficulty of identification between object categories. Our previous study confirmed that haptic and visual recognition of hand identity also activates EBA (Kitada et al. 2009). Presentation software (Neurobehavioral Systems) was used for recording responses in all tasks and for presenting objects in the visual conditions.

#### Task

In each rTMS session before and after cTBS, the participants used either vision or haptics to perform object recognition tasks. Participants were instructed to identify objects as soon and as accurately as possible by pressing one of the 4 buttons corresponding to exemplars with the left hand. Participants completed practice trials until their performance accuracy reached a correctness level of at least 70% in each sensory modality. This practice was conducted during the second session when the resting motor threshold was measured and before initiating the main experiment in each of the rTMS sessions. The total time for the poststimulation tasks was <40 min, since the aftereffect of cTBS has been shown to last for at least 50 min (Huang et al. 2005; Wischnewski and Schutter 2015). At the end of each stimulation session, participants filled out a questionnaire to measure their levels of attention, fatigue, pain, and sleepiness on a 10-point scale [e.g. attention (1 = no distraction; 10 = highest level of distraction)].

#### Visual object recognition

Participants were seated at approximately arm’s length away (≈70 cm) from a 24-inch LCD display (U2417H; Dell Inc., TX, USA) and watched monochrome photos of the objects that were used in the haptic task. Images were modified to minimize differences in size and perceived brightness (Fig. 1c). For each exemplar, 2 monochrome images taken from 2 different views (front and back) were presented. Object presentation was preceded and succeeded by a white-cross presentation. The stimuli and the white fixation cross subtended visual angles of ~9.8° and 1.7°. Each visual image was presented 6 times for a duration of 500 ms with 1500 ms ISI. For each pre_TMS and post_TMS phase, each stimulus was presented with 6 repetitions in 2 runs (3 categories × 4 exemplars × 2 views × 6 repetitions = 144 trials in total, 72 trials for each run). The order of images presented was pseudo-randomized. Each run lasted around 3 min, and the whole visual task took <10 min.

#### Haptic object recognition

Blindfolded participants were seated in front of a table and identified the presented objects with their right hand. We recorded the onset of hand exploration by placing objects on a wooden plate on top of a footswitch (RS27H; Olympus, Japan) that detected the initial force exerted by the participant’s hand (Fig. 1d). Objects were secured on the plate with Velcro tape. The orientation of the presented objects was the same as in the previous study (Kitada et al. 2014). More specifically, hands and cars were pointed toward the participant. The center between the spout and handle of teapots was pointed toward the participant. In each trial, participants were given 2 auditory cues by the experimenter; they were asked to raise and keep their right hand over the object when they heard “Ready” and explore the object once they heard “Go.” The participants were asked to identify the exemplar within 10 s. Each stimulus was presented with 6 repetitions for each pre_TMS and post_TMS phase (i.e. 3 categories × 4 exemplars × 6 repetitions = 72 trials in each phase). The order of objects presented was pseudo-randomized. The number of trials for vision and haptics differed (144 vs. 72 trials) because our aim was not to directly compare behavioral performance between the 2 modalities. The haptic task took ~15 min to complete.

### Confirmatory data analyses

We conducted confirmatory analyses and exploratory analyses based on the preregistration. We analyzed performance accuracy (i.e. percent correct: PC), RT, and inverse efficiency scores (IES; Townsend and Ashby 1983) using IBM SPSS Statistics (v22.0, IL, USA). Although IES gives a summary of the findings by combining accuracy and RT (i.e. RT/PC), separate analyses on each of them are advised because of the variance increase (Bruyer and Brysbaert 2011). We tested a priori hypotheses using preregistered linear contrasts without multiple comparison corrections. This procedure is statistically valid (Keppel and Wickens 2004) and analogous to linear contrasts commonly used in functional MRI analyses. Nonparametric tests were conducted because data under some conditions violated the normality test assumptions (Shapiro–Wilk tests, see Results section).

To evaluate our predefined hypotheses, we calculated EBA-specific effects as follows. First, we calculated the effect of cTBS for each target region by subtracting the behavioral performance of the task before TMS (pre_TMS) from the task after TMS (post_TMS). Then we obtained the EBA-specific effect by subtracting the control-region stimulation effect (post_TMS − pre_TMS) from the EBA effect (post_TMS − pre_TMS). And finally, the EBA-specific *hand* effect was determined by comparing the EBA-specific effects of hands with the effects of cars. Likewise, we calculated the EBA-specific teapot effects by comparing the EBA-specific effects of teapots with the effects of cars. In other words, we defined the EBA-specific hand effect as the 3-way interaction effect for hands [(post_TMS − pre_TMS) × (EBA − vertex) × (Hand − Car)] and the EBA-specific teapot effect as the 3-way interaction effect for teapots [(post_TMS − pre_TMS) × (EBA − vertex) × (Teapot − Car)]. The EBA-specific hand effects and EBA-specific teapot effects were evaluated for each sensory modality.

#### Preregistered Hypothesis 1 (visual hand recognition)

We hypothesized that, because EBA in each hemisphere is associated with visual hand processing, rTMS of EBA would worsen performance on hand identification more than the identification of control objects (cars). Thus, we conducted a 1-tailed 1-sample Wilcoxon signed-rank test on the EBA-specific hand effect for each hemisphere. We then compared EBA-specific hand effects between the 2 hemispheres by conducting a Wilcoxon matched-pairs signed rank test. We expected the right hemisphere to show a stronger effect than the left hemisphere (Willems et al. 2010). We reported r as the effect size (Rosenthal 1991).

#### Preregistered Hypothesis 2 (haptic hand recognition)

We then tested whether rTMS over EBA would also worsen the performance of haptic hand recognition by conducting 1-tailed Wilcoxon sign-ranked tests on EBA-specific hand effects.

#### Preregistered Hypothesis 3 (tool recognition)

Because EBA is adjacent to and partially overlapped with tool representation, we hypothesized that rTMS over EBA would worsen the performance of teapot recognition in both sensory modalities. We tested this hypothesis by performing Wilcoxon sign-ranked tests on data in each sensory modality (1-tailed).

### Exploratory analyses

#### rTMS effect on object recognition

Although EBA is defined as a body-sensitive region, the same region is more sensitive to objects than scenes (Downing et al. 2006), which is similar to the response preferences of LO (Grill-Spector et al. 2001). In fMRI studies, object preference in LO was observed in haptics when compared with textured surfaces (Amedi et al. 2001; Stilla and Sathian 2008) and with baseline (James et al. 2002). Therefore, it is possible that rTMS over EBA worsens the performance of object recognition, regardless of the object category. To test this hypothesis, we collapsed the factor of object category and evaluated the 2-way interaction terms for hands [(post_TMS − pre_TMS) × (EBA − vertex), EBA object effect] by conducting 1-tailed Wilcoxon sign-ranked tests separately on vision and haptics (with Bonferroni correction)*.* Subsequently, we compared the EBA object effect between the right and left hemispheres by performing a 2-tailed Wilcoxon signed-rank paired test.

#### EF analysis

We estimated the strength of the induced EFs in the target regions during rTMS by constructing a computational model (Laakso et al. 2016). First, the cortical EFs were numerically calculated in individual MRI-based anatomical models (constructed from T1- and T2-weighted images) using the finite element method with first-order cubical elements (0.5 mm side length), which were assigned electric conductivity values as described in Laakso et al. (2018). The individual EF strengths were first evaluated on a surface 2 mm below the gray matter–CSF boundary, and the surface EFs were then mapped to the fsaverage brain template using FreeSurfer image analysis software (Dale et al. 1999; Fischl et al. 1999; Fischl and Dale 2000). Through this method, population-average EFs and their variability in standard brain space were calculated (Laakso et al. 2016). We tested our hypotheses that the estimated EF would be associated with the EBA-specific hand effect and EBA-specific teapot effect by conducting nonparametric correlation analyses (Spearman correlation).

## Results

### Individual fMRI functional localization

**Fig. 2:**
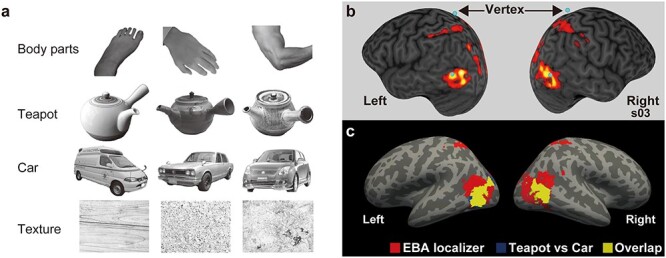
Functional localizers and target regions a) examples of stimuli used in fMRI localizer task. b) The EBA localizer of a representative participant (s03). The anatomical image and activity are shown in the participant’s native space. Target regions are shown as blue spheres. Activity was thresholded at the height (cluster-forming) level of *P* < 0.001 uncorrected for multiple comparisons. c) Group-mean activation shown by the EBA localizer (hands compared with the means of other stimuli) and tool-sensitive region (teapot vs. car). Activation is overlaid on a template surface (fsaverage) in MNI space. The statistical threshold for the spatial extent test was set at *P* < 0.05, FWE-corrected for multiple comparisons when the height threshold was set at *P* < 0.001 uncorrected (*n* = 26).

Target areas in the EBAs for each participant were identified with a visual localizer task. The mean peak coordinates of EBA were *x* = −48.6 ± 2.7, *y* = −73.4 ± 4.9, *z* = 1.8 ± 4.7 (mean ± SD) for left EBA and *x* = 52.2 ± 3.3, *y* = −66.2 ± 5.1, *z* = 0.3 ± 4.5 for right EBA, which were consistent with the previous studies (e.g. Peelen and Downing 2005a, 2005b; Zimmermann et al. 2018). Supplementary Table 1 shows the MNI coordinates for each participant, whereas Fig. 2b shows the target EBA region of 1 representative participant.

Figure 2c shows the group-mean activation by the EBA localizer and localizer of teapot-sensitive region (teapot vs. car). The conjunction analysis using an inclusive masking procedure (Rajaei et al. 2018; Kitada et al. 2021) confirmed significant overlap of activation between teapot vs. car and EBA localizer (Fig. 2c). We also confirmed the overlap of activation between EBA and LO in Supplementary Fig. 1.

As a supplementary analysis, we evaluated the hemispheric laterality of EBA, LO, and teapot-sensitive region. We flipped contrast images in the horizontal (right–left) direction and conducted a paired *t*-test between nonflipped and flipped contrast images (Kitada et al. 2006; Rajaei et al. 2018). However, none of the localized regions showed significant laterality effect.

### Confirmatory analyses

#### Visual hand recognition

**Fig. 3:**
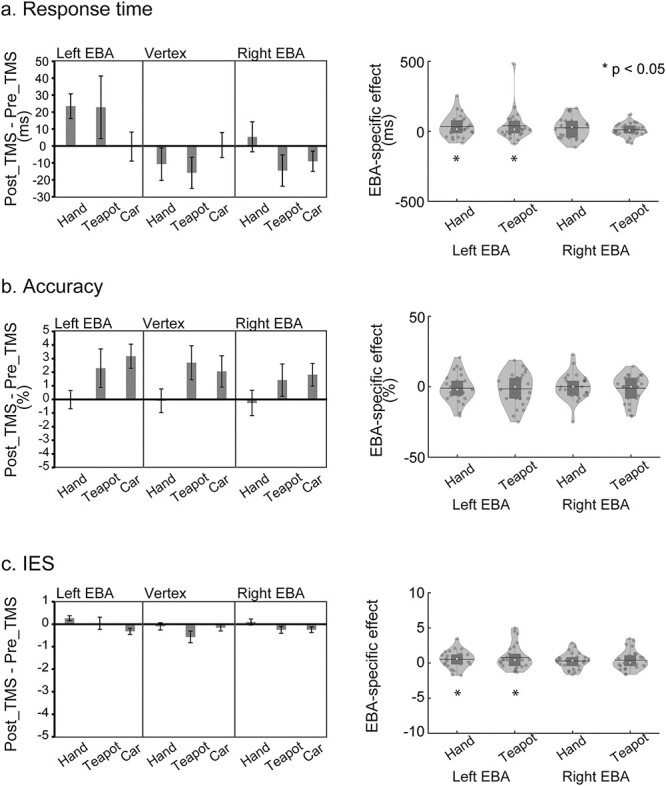
Visual hand and teapot effects bar graphs (left) indicate the intervention effects (post_TMS − pre_TMS phases) of the visual task. The inhibition after the rTMS application was indicated by positive values in RT a) and IES c) and by negative values in accuracy b). Cars were used as control objects. Error bars indicate SEM. Violin and boxplots on the distribution of EBA-specific hand effects and teapot (tool) effects are shown (right). Each dot represents the individual participant’s data. The white circle, horizontal line, and bar indicate the median, mean, and box plot, respectively. Asterisks indicate the results of 1-tailed 1-sample Wilcoxon signed-rank test.

Figure 3a (left) shows relative RTs, that is, RTs of the pre_TMS phase subtracted from the post_TMS phase. Group-mean data in the pre_TMS and post_TMS phases are available in Supplementary Table 2. Relative RTs for hands in left EBA were greater than those in the vertex, whereas relative RTs for cars (control) were comparable between the left EBA and vertex. To further examine this pattern, Fig. 3a (right) shows the EBA-specific hand effect, which was defined as the 3-way interaction [(post _TMS − pre_TMS) × (EBA − vertex) × (hand − car)] in the preregistration. If the TMS over EBA disturbed hand processing, the EBA-specific hand effect in RT (and IES) would become >0, whereas the same effect for accuracy would become <0. We used nonparametric tests because of outliers (*P*-values < 0.05, Shapiro–Wilk test).

To test Hypothesis 1, we performed a 1-sample nonparametric test (Wilcoxon signed-rank test) on EBA-specific hand effects for left and right EBA. This test on RT revealed that the EBA-specific hand effect was significantly >0 for left EBA (*P* = 0.019, 1-tailed, *r* = 0.41; Rosenthal 1991). In other words, the RT for hands relative to cars was more strongly affected by rTMS over left EBA than by rTMS over the vertex. Similarly, the same test on IES also revealed that the EBA-specific hand effect was significantly >0 for left EBA (*P* = 0.017, 1-tailed, *r* = 0.42) (Fig. 3c). No significant effect was observed for right EBA (*P-*values > 0.06, 1-tailed).

In the next test, we compared the laterality by comparing the EBA-specific hand effects between the left and right EBA. However, Wilcoxon signed-rank tests on RT and IES revealed no significant difference (*P*-values > 0.2, 2-tailed).

Figure 3b (left) shows the accuracy in the post_TMS phase, from which the accuracy in the pre_TMS phase was subtracted (relative accuracy). As compared with hands, the accuracy of other object categories appeared to increase after the post_TMS phase (Supplementary Table 3). As in RTs, we performed nonparametric tests (Wilcoxon signed-rank tests) on EBA-specific hand effects for left and right EBA (Fig. 3b, right). However, no significant effects were observed (*P-*values > 0.2, 1-tailed).

#### Visual teapot recognition

rTMS caused a similar pattern of change between teapots and hands in left EBA (Fig. 3a and Supplementary Tables 2 and 4). One-tailed Wilcoxon signed-rank test on RT revealed that EBA-specific teapot effect in vision was significantly >0 in left EBA (*P* = 0.041, *r* = 0.34). We also observed similar results for IES for left EBA (*P* = 0.046, 1-tailed, *r* = 0.33, Fig. 3c). In contrast, the accuracy showed similar patterns between teapots and cars (Fig. 3b and Supplementary Table 3). No significant effect was observed in the accuracy of left EBA (*P* > 0.2, 1-tailed). We also observed no significant EBA-specific teapot effect in right EBA (*P-*values > 0.1, 1-tailed).

We also compared the laterality by comparing the EBA-specific teapot effects between the left and right EBA. However, Wilcoxon signed-rank tests on RT and IES revealed no significant difference (*P-*values > 0.2, 2-tailed).

#### Haptic hand recognition

**Fig. 4:**
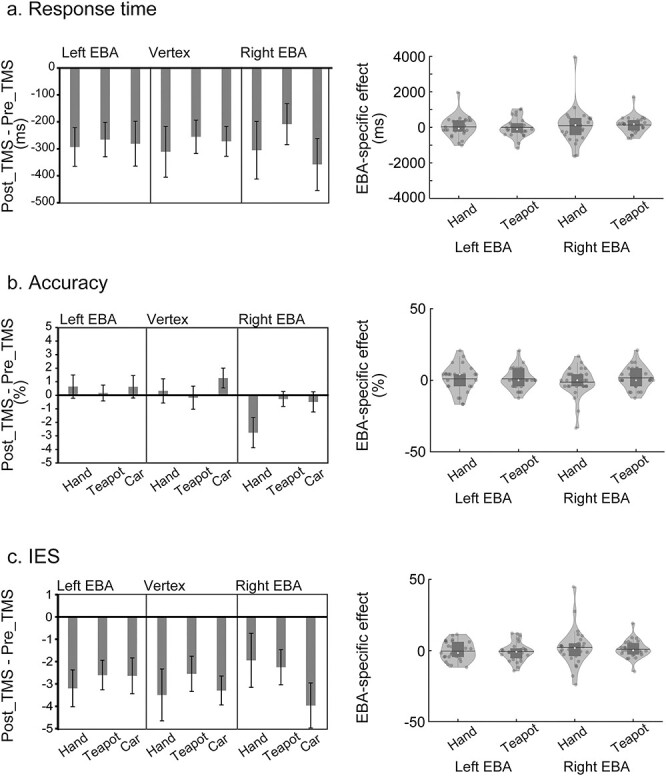
Haptic hand and teapot effects bar graphs (left) indicate the intervention effects (post_TMS − pre_TMS phases) of the haptic task. a, b, and c) indicate RT, accuracy, and IES, respectively. Error bars indicate SEM. Violin and boxplots (right) show the distribution of EBA-specific hand and teapot effects. No significant effect was observed.

Figure 4a (left) shows relative RTs for haptic object recognition. Relative RTs (post_TMS − pre_TMS) of all conditions showed negative values, indicating that the performance of object recognition was improved by the rTMS (Supplementary Tables 2–4 for both pre_TMS and post_TMS data). Unlike the vision patterns, however, RT patterns were similar between object categories and between stimulated brain regions. One-sample nonparametric tests (Wilcoxon signed-rank tests) on EBA-specific hand effects of RT for each hemisphere (Fig. 4a, right) showed no significant effect (*P-*values > 0.4, 1-tailed).

Figure 4b (left) shows the relative accuracy (i.e. accuracy in the post_TMS phase relative to the pre_TMS). Most of the conditions showed negligible changes after stimulation, whereas the hand recognition accuracy for right EBA appeared to decrease after the stimulation. However, 1-sample nonparametric tests (Wilcoxon signed-rank tests) on EBA-specific hand effects (Fig. 4b and c, right) showed no significant effect either for accuracy or for IES (*P-*values > 0.3, 1-tailed).

#### Haptic teapot recognition

We observed no clear rTMS effect on haptic recognition of teapots (Fig. 4). We performed 1-sample Wilcoxon signed-rank tests on EBA-specific teapot effects (Fig. 4, right). However, no significant effects were revealed by tests on either RT, accuracy, or IES (*P-*values > 0.09, 1-tailed).

#### Supplementary analysis

As no significant haptic effect was observed, we examined whether the rTMS effect varied over time. We analyzed RT, sIES, and accuracy of the haptic task across 6 repetitions of trials. One-sample Wilcoxon signed-rank tests were conducted on EBA-specific teapot effects, revealing significant effects on RT and IES only in the first repetition for right EBA (*P-*values < 0.05, 1-tailed test, Bonferroni-corrected over 6 repetitions). No other significant effect was observed.

#### Does the difference in difficulty among object categories explain preferences for hands and teapots?

The premise of the analyses above was that behavioral performance in the pre_TMS phase is comparable across the regions and across object categories (Supplementary Tables 2–4). We confirmed that behavioral performance in the pre_TMS phase was comparable across target regions in RT, accuracy, and IES (*P-*values > 0.09, Friedman’s ANOVA by Ranks). However, behavioral performance in the pre_TMS phase differed among object categories (Table 1). Wilcoxon matched-pair signed-rank tests revealed significantly slower RT and greater IES for visually perceived teapots than for visually perceived cars (*P* = 0.032 for RT and *P* = 0.028 for IES, 2-tailed).

**Table 1:** Pre-stimulation behavioral performance mean object scores.

	**Vision**			**Haptics**		
	**Hand**	**Teapot**	**Car**	**Hand**	**Teapot**	**Car**
RT (ms)	782 ± 17	807 ± 18	772 ± 14	3,439 ± 166	2,760 ± 158	2,970 ± 148
Accuracy (%)	92.5 ± 0.8	87.7 ± 1.4	90.9 ± 0.7	97.3 ± 0.6	98.4 ± 0.4	97.7 ± 0.7
IES	8.5 ± 0.2	9.3 ± 0.3	8.5 ± 0.2	35.5 ± 1.8	28.2 ± 1.7	30.5 ± 1.5

Pre_TMS task scores were calculated as the average of 3 sessions. IES was calculated as RT/accuracy. Data are the group object means ± SEM.

Then does the slower RT for visually perceived teapots relative to that for cars explain the significant teapot effects (see Fig. 3a)? To address this question, we calculated the difference of RT and IES between teapots and cars in the pre_TMS phase and examined correlations with the EBA-specific teapot effect. However, no significant correlation was observed (*P-*values > 0.3, 2-tailed). As a supplementary analysis, a simple linear regression analysis with EBA-specific teapot effect as the dependent variable and with the difference in RT between teapots and cars (in the pre_TMS phase) as an independent variable showed that the constant term was significantly >0 [*t*(24) = 2.11, *P* = 0.023 1-tailed], whereas the same analysis on IES showed a tendency toward significance [t(24) = 1.70, *P* = 0.051 1-tailed]. This result confirms the presence of an EBA teapot effect despite differences in task difficulty.

### Exploratory analyses

We then conducted analyses that were not specified in the preregistration (exploratory analyses). Preregistration allows us to conduct exploratory analyses though the results are considered to be more tentative than confirmatory (Wagenmakers et al. 2012). Unlike in the confirmatory analysis, we controlled FWE using Bonferroni corrections.

#### EF analysis

**Fig. 5:**
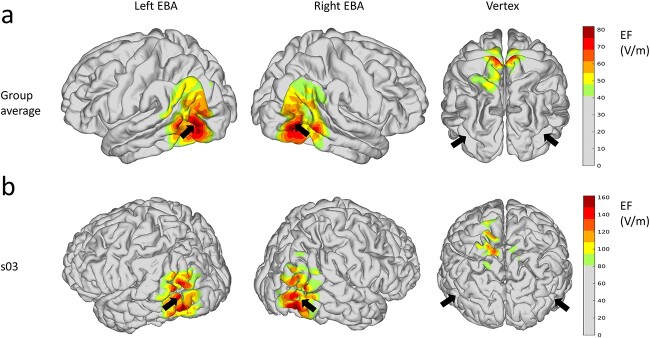
EF analysis a) group average of the EF strength shown on the template brain (fsaverage) when rTMS was applied over the left EBA, right EBA, and vertex. Black arrows show the locations of the left and right EBA (the center of gravity of hand-sensitive activation in Fig. 2). EF strengths weaker than 50% of the maximum are shown in gray. b) The EFs on the brain of a representative subject (s03).

We estimated the EFs that were produced by cTBS in the brain, as in our previous study (Laakso et al. 2016). EF modeling revealed that the highest group-average EFs were located in regions in and around EBA, whereas the EF was spread over LOTC (Fig. 5). We extracted maximum induced EF strength in each EBA (over a 1 cm radius sphere centered at the individual target coordinates). Wilcoxon matched-pair sign-ranked tests on maximum induced EF showed no significant difference between the left and right EBA (*P* > 0.7, 2-tailed). We then examined the correlations between EF values and performance scores (RT, accuracy, and IES) to test whether the simulated EF can explain the EBA-specific hand and teapot effects (with Bonferroni correction over 4 tests, 2 EBAs × 2 sensory modalities). However, no significant effect was observed (*P-*values > 0.1, Spearman correlation).

#### Does the region in and around EBA represent a preference for objects?

In the confirmatory analyses, neither EBA-specific hand effects nor teapot effects in haptics were significant. As in previous studies, we also confirmed that EBA overlaps with LO (Supplementary Fig. 1). Moreover, the induced EF was shown around EBA. Thus, rTMS over EBA can also worsen the performance of haptic recognition of all object categories, which might mask EBA-specific hand and teapot effects.

**Fig. 6:**
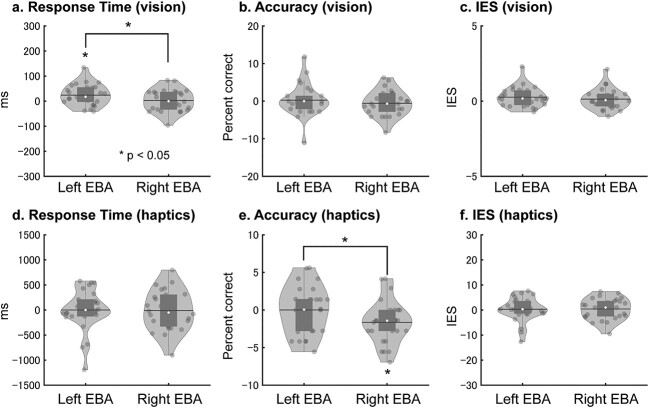
EBA-specific object effects for RT in vision a), accuracy in vision b), IES in vision c), RT in haptics d), accuracy in haptics e), and IES in haptics f) are shown. EBA-specific object effects are calculated as the 2-way interaction of phase and region [(post_TMS − pre_TMS) × (target region − vertex)]. Thus, if the TMS over EBA disturbed object processing, the EBA object effect in RT and IES would become >0, whereas the same effect for accuracy would become lower than 0. An asterisk indicates the significant results of nonparametric tests [Wilcoxon sign-ranked tests (Bonferroni-corrected over 4 tests) and Wilcoxon matched-pair sign-ranked tests].

To examine this possibility, we collapsed the factor of object category and evaluated the region-by-phase (time) interaction (EBA-specific object effect) for RT, accuracy, and IES (Fig. 6). This procedure is justified because no significant difference among object categories was observed in haptics (*P-*values > 0.08, 2-tailed Wilcoxon signed-rank tests). If the TMS over EBA disturbed object processing, the EBA-specific object effect in RT (and IES) would become >0, whereas the same effect for accuracy would become lower than 0. We performed 1-tailed Wilcoxon sign-ranked tests on the EBA-specific object effect of RT with Bonferroni correction for multiple comparisons (over 4 tests, 2 EBAs × 2 sensory modalities). We used 1-tailed tests based on the assumption in the preregistration that cTBS causes inhibitory effects. This test revealed that the left EBA-specific object effect of RT in vision was significantly >0 (*P* = 0.026 Bonferroni-corrected, *r* = 0.49, Fig. 6a). The same nonparametric tests on accuracy (with Bonferroni correction) showed that the right EBA-specific object effect in haptics was significantly lower than 0 (*P* = 0.026, *r* = 0.49, Fig. 6e). No significant effect was observed for IES (*P-*values > 0.1, Fig. 6c and f).

To examine the laterality of the effect, we compared the EBA-specific object effect between the left and right EBA. Wilcoxon matched-pair sign-ranked tests on RT showed that the EBA-specific object effect in vision was greater for the left than the right hemisphere (*P* = 0.025 2-tailed, Fig. 6a). Wilcoxon matched-pair sign-ranked tests on accuracy showed that the EBA-specific object effect in haptics was significantly lower for right than left EBA (*P* = 0.036 2-tailed, Fig. 6e). No significant effect was observed for IES (*P-*values > 0.08, 2-tailed, Fig. 6c and f).

#### Post-session questionnaire

None of the participants reported lasting adverse side effects after the stimulation sessions. Table 2 shows scores of attention, fatigue, pain, and sleepiness after rTMS. Overall, the participants reported low scores (<5 out of 10), though there were some differences between target regions. More specifically, although pain ratings were low (1–2), Wilcoxon signed-rank test on scores of pain showed that EBA stimulations were significantly more painful than vertex stimulations (left EBA, *P* = 0.008; right EBA, *P* = 0.002, 2-tailed). No other test showed a significant difference (*P-*values > 0.1). We compared pain scores of both hemispheres using Wilcoxon matched-pair signed-rank tests. However, no significant difference was observed between the 2 hemispheres (*P* = 0.67, 2-tailed).

**Table 2:** Questionnaire scores after each intervention.

	Left EBA	Right EBA	Control (vertex)
Attention	3.5 ± 0.5	3.3 ± 0.4	3.1 ± 0.4
Fatigue	3.1 ± 0.4	2.9 ± 0.4	2.6 ± 0.4
Pain	2.0 ± 0.3	1.9 ± 0.2	1.1 ± 0.1
Sleepiness	3.0 ± 0.4	3.4 ± 0.4	3.2 ± 0.4

TMS effects scored on a 10-point scale (e.g. 1: no pain; 10: highest pain). Data show the group means ± SEM.

Finally, we examined the correlations between cTBS pain scores and performance scores (RT, accuracy, and IES) to test whether the pain felt by participants could explain the observed effects. However, there were no significant correlations between the performance scores and pain scores (*P-*values > 0.1, Spearman correlation).

## Discussion

The present study examined the effect of cTBS over EBA to examine the functional relevance of this region in the bisensory representation of hands and teapots. Preplanned comparisons showed that rTMS over EBA delayed RT on visually perceived hands and teapots. Exploratory analyses showed that rTMS over right EBA reduced the performance accuracy of haptically perceived objects more than rTMS over left EBA. In contrast, rTMS over left EBA delayed the RT of visually perceived objects more significantly than rTMS over right EBA.

### rTMS over EBA worsened the visual identification of hands and teapots

In the present study, cTBS over EBA caused worse identification performance for the visually perceived hands than control objects (cars). Previous studies used online rTMS over EBA (Urgesi et al. 2004; Pitcher et al. 2009, 2012). Using the confirmatory analyses, the present study extended these previous findings by showing that *offline* rTMS can also affect the hand-related processing of vision. This result indicates that cTBS over EBA can cause a short-term modulation of excitability in and around EBA and its associated network.

Another novel finding in the present study is that rTMS over EBA also delayed the processing of visually observed teapots. This effect was not explained by the difference in difficulty between teapots and control objects; no significant correlation was observed with performance in the pre_TMS phase. Therefore, it is unlikely that the task demand accounts for the EBA teapot effect. Previous fMRI studies have shown overlap between tool-sensitive and hand-sensitive activation in LOTC (Bracci et al. 2012; Bracci and Peelen 2013). Indeed, other fMRI studies also showed greater responses to visually observed tools than other objects, such as cars in EBA (Downing et al. 2006; Kitada et al. 2014). The present study supports these neuroimaging findings by showing that regions in and around EBA are functionally relevant for processing tools as well as body parts.

Our results are consistent with previous fMRI studies that demonstrated a close interaction between hands and tools in left LOTC (Bracci and Peelen 2013; Maimon-Mor and Makin 2020). For example, Bracci and Peelen (2013) examined a hand-selective region in left LOTC that responded more strongly to objects that can replace the hand as the end effector of the action (object effectors, e.g. rackets and combs) than other objects (e.g. graspable objects and musical instruments). Thus, because teapots can also be considered a kind of effectors (e.g. because they function to pour tea into cups), rTMS over EBA may have delayed the RT required to identify exemplars of teapots.

We expected a stronger activation in the right than left EBA based on a previous study (Willems et al. 2010). However, we observed no such effect in the rTMS experiment. Our result is rather in accord with another finding that a region around left EBA is more sensitive to hands than whole body and other body parts, whereas no such pattern was observed in right EBA (Bracci et al. 2010).

### rTMS over EBA worsened the haptic object identification

Our exploratory analyses showed that rTMS over EBA affects haptic object identification (performance averaged over object categories) more strongly than stimulation over the vertex. In addition, the laterality of the rTMS intervention effects differed; rTMS over left EBA affected visual object identification, whereas rTMS over right EBA affected haptic object identification.

This result indicates that rTMS over EBA modulates multisensory object processing. In our functional localizer, portions of EBA overlapping LO showed a preference for objects over textures in accordance with previous fMRI studies (Supplementary Fig. 1). A previous study showed that rTMS over LO disrupts the visual recognition of familiar objects more strongly than that of scenes (Mullin and Steeves 2011). Another study found that LO was functionally relevant to the Müller-Lyer illusion that occurs in both vision and touch using low-frequency rTMS (Mancini et al. 2011). Here, we extended the previous finding by showing for the first time that rTMS over EBA can temporarily impair the haptic recognition of familiar 3D objects.

To evaluate object preference in EBA, we compared the effects of intervention in EBA and vertex. Although the rating of pain because of peripheral stimulation differed between EBA and vertex, the pain ratings for all target regions were low (2 out of 10) and no significant correlation was found between the pain rating and behavioral performance. Moreover, we found a significant laterality in the EBA-specific object effect. Because left and right EBA showed comparable peripheral stimulation effects (Table 2), it is unlikely that our result can simply be explained away by the difference in peripheral stimulation. Likewise, it is unlikely that the task demand itself explains the result, because the behavioral performance before rTMS application was comparable between target regions. We assigned alphabet labels to each object and the verbalization process would be canceled out by comparing one object category to control objects (cars). Finally, though mean PC was high (over 97%), we used nonparametric analysis, which is less affected by skewness of data. Thus, the ceiling effect is unlikely to explain the observed difference.

rTMS involves stimulation from outside of the skull and can modulate cortical regions beyond the target point. Our EF analysis indicates that the rTMS affects a large region of the LOTC. Moreover, EBA is also partially overlapped with LO; the region can show a greater response to objects than textures (Bracci et al. 2012; Bracci and Peelen 2013). Previous fMRI studies have demonstrated that LO is also sensitive to haptically perceived objects (Amedi et al. 2001, 2002; James et al. 2002). Thus, it is possible that rTMS affected portions of LOTC beyond EBA, including part of LO, and that these effects disrupted multisensory object processing. This could have masked the hand-preference response in haptics.

This finding appears to be inconsistent with those of a previous rTMS study (Kassuba et al. 2014) and a lesion study (Snow et al. 2015). More specifically, Kassuba et al. (2014) applied offline low-frequency rTMS to left LO immediately before participants performed a delayed-match-to-sample task. However, rTMS failed to affect the performance of object discrimination regardless of whether haptics or vision was used. Moreover, Snow et al. (2015) demonstrated that a patient with extensive lesions, including lesions to the bilateral LO, was able to identify objects by touch but not by vision (Snow et al. 2015).

One possible explanation for this discrepancy is that regions in LOTC may be nodes of the brain network that is essential for visual object recognition. For instance, visual imagery of haptically perceived objects may contribute to more accurate and speedy identification (Lederman et al. 1990) and is associated with activity of the occipital lobe (Sathian et al. 1997). Thus, the rTMS over EBA disrupts the visual imagery of the objects, which leads to the reduction of performance for haptic object recognition. If haptic activation of LOTC reflects visual imagery, then the effects of rTMS over EBA on haptic object processing and visual object processing would be similar. However, rTMS to EBA differentially affected haptic and visual object recognition. Therefore, it is not clear whether only visual imagery during the haptic object recognition tasks can explain the TMS effect on haptic object recognition. Rather, it is more reasonable to assume that regions in LOTC play critical roles in the object recognition of haptics, as well as vision. For instance, it is possible that the brain network for haptic object recognition can adapt to lesions in LOTC through the long-term plastic change of the network.

As mentioned above, the laterality of the rTMS intervention effect differed between vision and touch; TMS to left EBA affected visual object recognition more strongly than TMS to right EBA. This result suggests different contributions of left and right LOTC to visual object recognition. A few neuroimaging and brain stimulation studies showed laterality effects of LO (Large et al. 2007; Bona et al. 2014). For instance, as compared with the visual object matching task, the visual object naming task resulted in more activity in left LO (Large et al. 2007). Indeed, a lesion over the left middle temporal gyrus can cause impairment in the naming of manipulable objects (Campanella et al. 2010). However, it is unclear if this explains the difference between vision and touch because touch would also involve naming objects. Rather our result is consistent with previous findings that the visual hand and tool representation were lateralized to the left hemisphere (Bracci et al. 2010, 2012; Peelen et al. 2013). It is possible that visual processing of hands and teapots were affected by rTMS, which resulted in the decrease of mean performance across object categories. In contrast, the finding that right rTMS interventions affected haptics more strongly than left rTMS interventions might be related to the right hemisphere dominance for haptic processing of microspatial properties (shape, and orientation, Harada et al. 2004; Kitada et al. 2006).

### Limitations and interpretational issues

There are 4 limitations worth noting. First, although we designed our experiments to cause focal stimulation of EBA, the simulation of EFs showed that rTMS can also affect areas beyond EBA. Moreover, such a remote effect could explain the observed pattern of temporal impairment. This point needs to be further validated in future studies that measure the activity after rTMS (e.g. Kassuba et al. 2014). Second, there is individual variability in cTBS effect (Hamada et al. 2013; Hordacre et al. 2017); cTBS suppresses MEP amplitude for high baseline MEP variability (Hordacre et al. 2017). It is necessary to examine the effect of rTMS on haptic object recognition using different stimulation protocols in future studies. Third, we did not include the texture as a control when examining object preferences (e.g. James et al. 2002; Amedi et al. 2010). This is partly because tactile texture recognition is more accurate and substantially faster than recognition of an object’s shape (e.g. Lederman and Klatzky 1997), and we could not match task difficulty in the pilot experiment. Although object preference in LO was compared with the baseline in some previous studies (James et al. 2002), it is necessary to compare the TMS effect on objects with the TMS effect on textures. Finally, IES in haptics in exploratory analyses was not significant, possibly because IES is more insensitive to statistical tests than accuracy and RT because of variance increase (Bruyer and Brysbaert 2011). Thus, this result should be replicated in the future study.

In conclusion, the present study showed that cTBS over left EBA temporarily impaired visual recognition of hands and teapots. In contrast, cTBS over EBA did not cause a category-specific impairment in haptic object recognition. The simulation of the induced EFs confirmed that the cTBS affected regions including EBA. These results indicate that the LOTC is functionally relevant for visual hand and tool processing and that the rTMS over this region may differently affect object recognition between the 2 sensory modalities.

## Supplementary Material

Final_suppl_Atilgan_offlineTMS_ms_2023March31RK_texcom_tgad005

## Data Availability

The data sets generated during and/or analyzed during the current study will be available at OSF (https://osf.io/xudj6) after the publication.

## CRediT authors statement

Hicret Atilgan (Data curation, Formal analysis, Investigation, Project administration, Visualization, Writing—original draft, Writing—review & editing), J.X. Janice Koi (Investigation), Ern Wong (Investigation), Ilkka Laakso (Formal analysis, Software, Supervision, Visualization), Noora Matilainen (Formal analysis, Software, Visualization), Achille Pasqualotto (Conceptualization), Satoshi Tanaka (Conceptualization, Supervision), S. H. Annabel Chen (Writing—review & editing), and Ryo Kitada (Conceptualization, Formal analysis, Funding acquisition, Investigation, Project administration, Supervision, Validation, Visualization, Writing—original draft, Writing—review & editing)
