# Supplementary material for: Functional relevance of the extrastriate body area for visual and haptic object recognition: a preregistered fMRI-guided TMS study
Source: Cereb Cortex Commun. 2023 Apr 13;4(2):tgad005. doi: 10.1093/texcom/tgad005 (PMC10176024; doi:10.1093/texcom/tgad005)
Supplement: Final_suppl_Atilgan_offlineTMS_ms_2023March31RK_texcom_tgad005 [file final_suppl_atilgan_offlinetms_ms_2023march31rk_texcom_tgad005.docx]

**Supplementary Information**

**Functional relevance of the extrastriate body area for visual and haptic object recognition: a preregistered fMRI-guided TMS study**

Hicret Atilgan, Janice JX Koi, Ern Wong, Ilkka Laakso, Noora Matilainen, Achille Pasqualotto, Satoshi Tanaka, Annabel S.H. Chen, Ryo Kitada^*^

**^*^Corresponding author:** Ryo Kitada, Graduate School of Intercultural Studies, Kobe University, 1 -2-1 Tsurukabuto, Nada Ward, Kobe, Hyogo 657-0013, Japan; Tel.: +81-78-803-7423; E-mail: [ryokitada@port.kobe-u.ac.jp](mailto:ryokitada@port.kobe-u.ac.jp)

**Supplementary Table 1 MNI coordinates of individual EBA.**

|  | **Left EBA** | | | | **Right EBA** | | | |
| --- | --- | --- | --- | --- | --- | --- | --- | --- |
| ID | x | y | z | Z value | x | y | z | Z value |
| s01 | -50 | -74 | 8 | 7.09* | 52 | -66 | 14 | 3.14* |
| s02 | -44 | -78 | 4 | 6.4* | 46 | -84 | 0 | 5.65* |
| s03 | -52 | -68 | 4 | Inf | 52 | -68 | 0 | Inf |
| s04 | -44 | -80 | 0 | 5.38 | 56 | -66 | -2 | 4.41 |
| s05 | -50 | -64 | 10 | Inf | 54 | -66 | 0 | 5.63 |
| s06 | -50 | -72 | 8 | 4.72* | 50 | -68 | 2 | 4.64* |
| s07 | -52 | -72 | 10 | 6.05* | 52 | -68 | 0 | 3.54* |
| s08 | -48 | -78 | 0 | 3.99 | 56 | -58 | 8 | 3.99 |
| s09 | -48 | -80 | 4 | Inf | 56 | -64 | -4 | 7.09 |
| s10 | -50 | -74 | 8 | Inf | 56 | -64 | -4 | Inf |
| s11 | -50 | -72 | -2 | Inf | 56 | -66 | 2 | Inf |
| s12 | -48 | -72 | 2 | Inf | 54 | -66 | -2 | Inf |
| s13 | -44 | -76 | -4 | Inf | 48 | -74 | 0 | Inf |
| s14 | -46 | -74 | -2 | Inf | 46 | -72 | -4 | Inf |
| s15 | -44 | -78 | 4 | Inf | 58 | -58 | -2 | 6.99 |
| s16 | -50 | -76 | 0 | 5.56 | 52 | -64 | 12 | 4.38 |
| s17 | -46 | -76 | -4 | Inf | 52 | -66 | -2 | Inf |
| s18 | -46 | -78 | 2 | Inf | 52 | -60 | -2 | 7.05 |
| s19 | -50 | -72 | -2 | Inf | 54 | -64 | 0 | Inf |
| s20 | -48 | -74 | -4 | Inf | 50 | -64 | 0 | Inf |
| s21 | -50 | -74 | -4 | Inf | 50 | -66 | 0 | Inf |
| s22 | -50 | -72 | 0 | Inf | 50 | -68 | 2 | Inf |
| s23 | -50 | -76 | -2 | Inf | 56 | -66 | -2 | 5.84 |
| s24 | -50 | -70 | -2 | Inf | 50 | -68 | 0 | Inf |
| s25 | -54 | -58 | 0 | Inf | 56 | -62 | -4 | Inf |
| s26 | -50 | -70 | 10 | Inf | 48 | -64 | 0 | Inf |
| Mean | -48.6 | -73.4 | 1.8 |  | 52.2 | -66.2 | 0.3 |  |
| SD | 2.7 | 4.9 | 4.7 |  | 3.3 | 5.1 | 4.5 |  |

Inf indicates that the value exceeds 8.0. These spatial coordinates were identified by the linear contrast of hands against the mean of the object categories. When this contrast failed to reveal activity in the EBA, we conducted the same analysis without teapots, because tools have comparable activity to body parts (indicated by asterisks).

**Supplementary Table 2 Mean response time and standard errors of the mean (SEM)**

| **Region** | **LEBA** |  |  | **Vertex** |  |  | **REBA** |  |  |
| --- | --- | --- | --- | --- | --- | --- | --- | --- | --- |
|  | **Hand** | **Teapot** | **Car** | **Hand** | **Teapot** | **Car** | **Hand** | **Teapot** | **Car** |
| **Vision (pre_TMS phase)** | | | | | | | | | |
| Mean | 781 | 788 | 776 | 795 | 830 | 771 | 771 | 803 | 769 |
| SEM | 19 | 21 | 17 | 21 | 26 | 16 | 13 | 15 | 12 |
| **Vision (post_TMS phase)** | | | | | | | | | |
| Mean | 804 | 811 | 776 | 784 | 814 | 771 | 777 | 788 | 760 |
| SEM | 22 | 22 | 17 | 20 | 27 | 16 | 17 | 14 | 12 |
| **Haptics (pre_TMS phase)** | | | | | | | | | |
| Mean | 3420 | 2754 | 3022 | 3458 | 2763 | 3022 | 3438 | 2762 | 2867 |
| SEM | 204 | 177 | 198 | 189 | 155 | 168 | 206 | 196 | 160 |
| **Haptics (post_TMS phase)** | | | | | | | | | |
| Mean | 3127 | 2489 | 2741 | 3147 | 2508 | 2750 | 3133 | 2554 | 2509 |
| SEM | 192 | 151 | 157 | 177 | 149 | 166 | 179 | 180 | 182 |

Response times are presented in milliseconds.

**Supplementary Table 3** **Mean accuracy and standard errors of the mean (SEM)**

| **Region** | **LEBA** |  |  | **Vertex** |  |  | **REBA** |  |  |
| --- | --- | --- | --- | --- | --- | --- | --- | --- | --- |
|  | **Hand** | **Teapot** | **Car** | **Hand** | **Teapot** | **Car** | **Hand** | **Teapot** | **Car** |
| **Vision (pre_TMS phase)** | | | | | | | | | |
| Mean | 92.5 | 87.9 | 90.3 | 92.8 | 86.0 | 90.3 | 92.3 | 89.3 | 92.1 |
| SEM | 1.3 | 1.5 | 1.3 | 1.1 | 2.0 | 1.1 | 0.9 | 1.3 | 0.9 |
| **Vision (post_TMS phase)** | | | | | | | | | |
| Mean | 92.4 | 90.1 | 93.4 | 92.6 | 88.6 | 92.3 | 92.0 | 90.6 | 93.9 |
| SEM | 1.2 | 1.5 | 1.1 | 1.2 | 1.5 | 1.1 | 1.1 | 1.5 | 0.8 |
| **Haptics (pre_TMS phase)** | | | | | | | | | |
| Mean | 96.5 | 98.9 | 97.6 | 97.6 | 98.1 | 97.4 | 97.8 | 98.2 | 98.1 |
| SEM | 1.1 | 0.4 | 0.9 | 0.7 | 0.6 | 1.0 | 0.7 | 0.9 | 0.7 |
| **Haptics (post_TMS phase)** | | | | | | | | | |
| Mean | 97.1 | 99.0 | 98.2 | 97.9 | 97.9 | 98.7 | 95.0 | 97.9 | 97.6 |
| SEM | 1.1 | 0.4 | 0.6 | 0.5 | 0.6 | 0.7 | 1.3 | 1.2 | 0.6 |

Accuracies are presented in % correct.

**Supplementary Table 4** **Inversion efficiency (IES) score and standard errors of the mean (SEM)**

| **Region** | **LEBA** |  |  | **Vertex** |  |  | **REBA** |  |  |
| --- | --- | --- | --- | --- | --- | --- | --- | --- | --- |
|  | **Hand** | **Teapot** | **Car** | **Hand** | **Teapot** | **Car** | **Hand** | **Teapot** | **Car** |
| **Vision (pre_TMS phase)** | | | | | | | | | |
| Mean | 8.5 | 9.1 | 8.7 | 8.6 | 9.9 | 8.6 | 8.4 | 9.1 | 8.4 |
| SEM | 0.3 | 0.3 | 0.3 | 0.3 | 0.5 | 0.2 | 0.2 | 0.2 | 0.2 |
| **Vision (post_TMS phase)** | | | | | | | | | |
| Mean | 8.8 | 9.1 | 8.4 | 8.5 | 9.3 | 8.4 | 8.5 | 8.8 | 8.1 |
| SEM | 0.3 | 0.4 | 0.3 | 0.3 | 0.4 | 0.3 | 0.2 | 0.3 | 0.2 |
| **Haptics (pre_TMS phase)** | | | | | | | | | |
| Mean | 35.6 | 27.8 | 30.5 | 35.6 | 28.3 | 31.2 | 35.2 | 28.6 | 29.7 |
| SEM | 2.2 | 1.8 | 1.9 | 2.1 | 1.6 | 1.8 | 2.1 | 2.3 | 1.8 |
| **Haptics (post_TMS phase)** | | | | | | | | | |
| Mean | 32.4 | 25.2 | 27.9 | 32.2 | 25.7 | 27.9 | 33.2 | 26.3 | 25.7 |
| SEM | 2.2 | 1.6 | 1.6 | 1.8 | 1.6 | 1.7 | 2.1 | 2.0 | 1.9 |


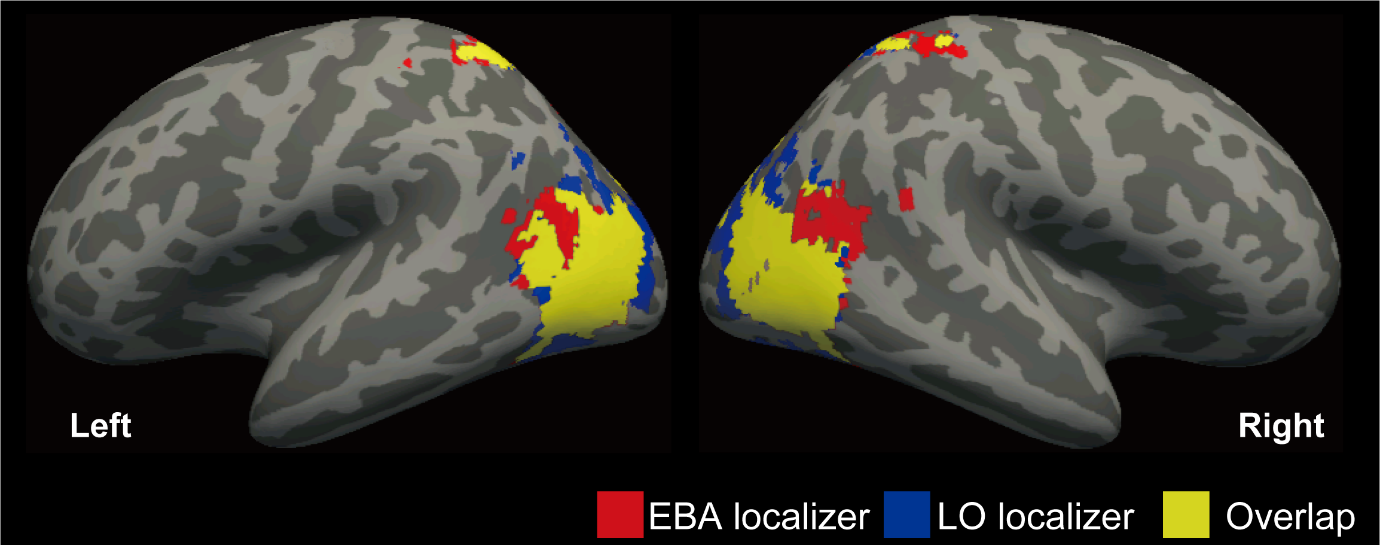


**Supplementary Figure 1 EBA and LO localizer**

Group-mean activation shown by the EBA localizer (hands compared to the means of other stimuli) and LO localizer (objects vs. texture). Activation is overlaid on a template surface (fsaverage) in MNI space. The statistical threshold for the spatial extent test was set at p < 0.05, family-wise error (FWE)-corrected for multiple comparisons when the height threshold was set at p < 0.001 uncorrected (n = 26).
